# Synthesis and Characterization of a New Molecularly Imprinted Polymer for Selective Extraction of Mandelic Acid Metabolite from Human Urine as a Biomarker of Environmental and Occupational Exposures to Styrene

**DOI:** 10.3390/polym15102398

**Published:** 2023-05-21

**Authors:** Murad. M. Qronfla, Bassem Jamoussi, Radhouane Chakroun

**Affiliations:** Department of Environmental Sciences, Faculty of Meteorology, Environment and Arid Land Agriculture, King Abdulaziz University, Jeddah 21589, Saudi Arabia

**Keywords:** molecularly imprinted polymer (MIP), solid-phase extraction (SPE), urine sample, mandelic acid (MA), urinary metabolite

## Abstract

4-Vinylpyridine molecularly imprinted polymer (4-VPMIP) microparticles for mandelic acid (MA) metabolite as a major biomarker of exposure to styrene (S) were synthesized by bulk polymerization with a noncovalent approach. A common mole ratio of 1:4:20 (i.e., metabolite template: functional monomer: cross-linking agent, respectively) was applied to allow the selective solid-phase extraction of MA in a urine sample followed by high-performance liquid chromatography–diode array detection (HPLC-DAD). In this research, the 4-VPMIP components were carefully selected: MA was used as a template (T), 4-Vinylpyridine (4-VP) as a functional monomer (FM), ethylene glycol dimethacrylate (EGDMA) as a cross-linker (XL), and azobisisobutyronitrile (AIBN) as an initiator (I) and acetonitrile (ACN) as a porogenic solvent. Non-imprinted polymer (NIP) which serves as a “control” was also synthesized simultaneously under the same condition without the addition of MA molecules. Fourier transform infrared (FT-IR) spectroscopy and scanning electron microscopy (SEM) were used to characterize the imprinted and nonimprinted polymer to explain the structural and morphological characteristics of the 4-VPMIP and surface NIP. The results obtained from SEM depicted that the polymers were irregularly shaped microparticles. Moreover, MIPs surfaces had cavities and were rougher than NIP. In addition, all particle sizes were less than 40 µm in diameter. The IR spectra of 4-VPMIPs before washing MA were a little different from NIP, while 4-VPMIP after elution had a spectrum that was almost identical to the NIP spectrum. The adsorption kinetics, isotherms, competitive adsorption, and reusability of 4-VPMIP were investigated. 4-VPMIP showed good recognition selectivity as well as enrichment and separation abilities for MA in the extract of human urine with satisfactory recoveries. The results obtained in this research imply that 4-VPMIP might be used as a sorbent for MA solid-phase extraction (MISPE), for the exclusive extraction of MA in human urine.

## 1. Introduction

It is crucial to keep an eye on the toxic chemical compounds and their metabolites in order to monitor what hazards they represent and what issues they could create given the rising worries about harmful substances such as styrene in the environment and workplace [1]. Styrene, commonly known as vinyl-benzene (C6H5CH=CH2), is a significant chemical in many manufacturing processes that is used to manufacture a variety of products such as polystyrene, latex paint and coatings, and synthetic rubber [2,3]. The use of products made with styrenes, such as packaging, electrical and thermal insulation, fiberglass, pipes, automobile components, and carpet backing, exposes many people to these chemicals on a daily basis [3]. Furthermore, styrene may also contaminate the food that has been packaged in polystyrene materials [4]. When it comes to absorbing styrene, inhalation is more effective than skin contact [5,6]. An amount of 3.5–76.2 g of styrene is typically present in the smoke from traditional cigarettes. Around 90% of the styrene in the body is predominantly converted to styrene-7,8-oxide (7,8-SO), which subsequently transforms to styrene glycol, can then be oxidized to produce MA, and then to phenyl glyoxylic acid (PGA) and is eliminated in the urine [6]. Eye irritation, respiratory symptoms, and nausea were seen after inhalation exposure to styrene (at concentrations >1600 mg/m^3^), whereas exposure to a dosage of >17 mg/m^3^ resulted in a reduction in color discrimination. As stated by the International Agency for Research on Cancer IARC (1985), it is considered to be “probably carcinogenic to humans” (category 2B) [4,5,7,8]. These metabolites represent the entire dosage taken from all exposure modes, including inhalation, skin, and oral absorptions, making them markers of internal exposure. As a result, these metabolites have seen widespread use in the biological monitoring of exposure to this solvent in the interest of protecting employees from developing occupational illnesses [9]. Moreover, owing to its non-invasive collection and abundance in metabolites, urine is a practical bio-fluid [10]. MA is a major metabolite of styrene with an excretion half-time of about 5–10 h in urine [2,11]. As stated by the American Congress of Governmental Industrial Hygienists (ACGIH), the threshold limit value (TLV) for MA metabolite as a result of exposure to styrene is 86 mg/m^3^ (20 ppm), and its Biological Exposure Index (BEI) is 400 mg/g creatinine in urine collected at the end of a work shift [5,6,11,12]. Chemical analysis requires precision equipment with pure samples completely isolated from interferents [13]. A crucial stage in the study of chemicals in biological samples is sample preparation. The most common cleaning method used to separate MA from urine samples is solid-phase extraction (SPE), which is preferred mainly to its ease, ability to save time, and use of a small quantity of solvent [14]. High-performance liquid chromatography–tandem mass spectrometry (HPLC-MS/MS) coupled with solid-phase extraction (SPE) was utilized by Ren J. Wei et al. (2020) to identify the metabolites of aromatic chemicals in urine samples. The linearity of MA was evaluated throughout a concentration range of 1–100 ng/mL and found to be high, with a correlation factor of >0.995 and a relative precision deviation (RSD) of 6.51%. Importantly, 80.1% of MA was recovered, and the method detection limit (MDL) was 0.092 ng/m [15]. Despite their advantages, SPE sorbents may not consistently exhibit strong selectivity for specific metabolites. In certain cases, matrix chemicals or interferents may be eluted alongside the target analytes and subsequently retained by the sorbent, leading to complications during the analytical process. To solve these issues, molecularly imprinted polymers (MIPs) can be employed as SPE sorbents, providing enhanced selectivity and enrichment to the target analyte from other similar compounds. The novel strategy is built on MISPE a hybrid of MIP and SPE [16]. MIPs are artificial polymers that have a chosen analyte imprinted on them. These polymers are created by covalent bonds. In this process, the initiator is meant to break down when exposed to light or heat to make free radicals. The polymerization process is started by free radicals, and the cross-linking agent brings together the template molecules and the functional monomers to make MIPs. To improve the effect of pre-polymerization, ultrasonic help may be used to speed up the process of dissolving and getting rid of dissolved oxygen, which can slow down the polymerization [4,10]. In our case, acidic organic solvents are used to get the target analyte out of the MIP after polymerization. Through chemical interactions, functional monomers with the same analyte or compounds with a similar structure may rebind to the 3D cavity left behind [14,17]. The noncovalent approach is the most common way to make MIPs for environmental analytes such as pure organic solvents and biological fluids because it is simple, the template molecule can be easily removed, and there are many commercially available functional monomers [17,18,19]. The vast majority of reported MIPs were made using bulk preparation techniques because of their many advantageous features. These include the ability to manufacture high-quality MIPs with little to no waste and no costly or complicated equipment. Then, the bulk polymer monolith is crushed, powdered, and sieved to produce irregularly shaped particles, mostly in the 25–100 m size range. After that, an elution process is carried out to remove tiny particles [20,21]. In 1973, Wulff and his team made a covalently bound MIP to separate racemic chemicals. In 1988, Mosbach et al. synthesized the first MIP with noncovalent interactions for L-phenylamine compounds. To restrict the number of experimental MIPs to be generated and evaluated, a computational technique such as response surface design attributes (RSDA), can be employed to assist in determining the template/monomer/cross-linker mole ratio [17,21,22]. In the present study, we utilized a conventional molar ratio of 1:4:20 to produce 4-vinylpyridine molecularly imprinted polymers (4-VPMIPs) of MA. These proportions have been previously shown to exhibit optimum values for imprinting factor, adsorption capacity, and binding efficiency [23,24]. The maximum adsorption capacity and binding kinetics of the MIP and non-imprinted polymer (NIP) were comparatively assessed through binding experiments. The study also investigated the binding selectivity and specificity of 4-VPMIP for MA and urine metabolites phenylglyoxylic acid (PGA) and hippuric acid (Hip) as competitive adsorption molecules. Furthermore, this study thoroughly examined the reusability of the 4-VPMIP to gain a deeper understanding of its capabilities.

Functional group compatibility is the strategy used to choose the functional monomer. Accordingly, basic functional monomers (such as 4-VP) are recommended for templates containing an acidic group (such as MA), while acidic functional monomers (such as Methacrylic acid; MAA) are employed to target bases [25]. Templates with active groups such as carboxyl and hydroxyl have been shown to improve MIP efficiency. Designing and building a high-performance MIP would be simplified by the presence of highly polar groups since more stable molecular combinations would be the result. Even though hydrogen bonds lead to positional accuracy, saturation, and strength, polymers that form them with functional monomers may have great specificity and affinity. For these reasons, 4-VP (base) was chosen as a monomer that can form a hydrogen bond with MA (N … OH). The ease of imprinting and extracting analytes shows that these noncovalent bonds are likely to happen. Larger templates are not as rigid, so they do not make well-defined binding cavities when they are imprinted. Because of this, most routine MIPs use small organic molecules as templates [23,26,27].

During the polymerization process, porogenic solvents are used to dissolve all of the ingredients used to make MIPs. This keeps the interaction between MA and 4-VP stable and helps create the porous structure of the 4-VPMIP. Additionally, the polarity of the porogen might influence interactions between the MA and 4-VP. It has an impact on an MIP’s adsorption characteristics, particularly in noncovalent interactions. A non-polar or less polar organic porogen, such as ACN, is employed to provide high printing efficiency for a noncovalent imprinting process. Hence, the adsorption and morphological characteristics of the produced polymer will depend on the kind of porogen or solvent utilized. Since ACN can dissolve all of the polymerization’s ingredients and has a low polarity (0.460), it was thought that it would make an MIP with a high imprinting efficiency. Water, on the other hand, might be the best solvent if hydrophobic forces are used to make the complex form [26,27].

The purpose of a cross-linker is to stabilize the imprinted binding site to create a cross-linked stiff polymer, arrange the functional monomers over template molecules in the polymerization process (control the morphology of the polymer matrix), and provide mechanical stability to the polymer matrix. For the recognition sites to remain stable, a cross-linker concentration of 80% is required. In this case, the 4-VPMIP may be able to “recognize” the original material because its functional groups are retained in an appropriate arrangement for rebinding the analyte after its removal, thanks to the microcavities’ highly cross-linked structures that preserve a 3D structure sequence similar in shape and chemical functionality to that of the template. Lastly, the cross-linker should make polymers that are stable at high temperatures and under stress, have good porosity, and are easy to create [26,27]. For these features, EGDMA was selected as a cross-linker.

**Figure 1:**
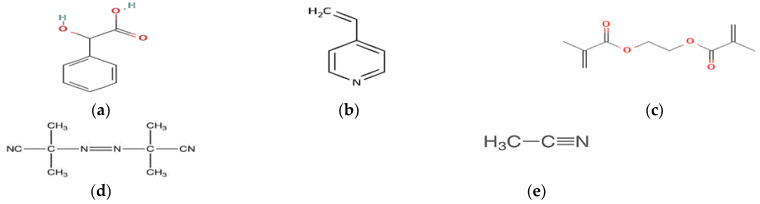
The molecular structures of MA-MIP components. (**a**) MA; C_6_H_5_CH(OH)CO_2_H; (**b**) 4-VP; CH₂CHC₅H₄N; (**c**) EGDMA; CH2=C(CH3) COOCH2CH2OCOC(CH3)=CH2; (**d**) AIBN; (CH_3_)_2_(CN)CN=NC(CN)(CH_3_)_2_; and (**e**) ACN; CH₃CN.

An initiator is a chemical that promotes rapid polymerization by starting the formation of new polymer chains. In comparison to the monomer, they are typically utilized at low amounts (1 weight percent or 1 mole percent of the total mass) [26,28]. If the azoinitiator (AIBN) is heated, it breaks down into carbon-centered radicals, which might initiate polymerization by adding a vinyl group (CH=CH_2_) to the monomer [23,26]. To speed up the growth of monomers and make sure that polymerizations can happen again, it is recommended to remove all dissolved oxygen from monomer solutions by ultrasonication or by sparging the monomer solution with an inert gas (such as N_2_) [26]. The chemical structures of all optimum components used in the 4-VPMIP synthesis are shown in Figure 1.

To extract gliclazide (GCZ) from human plasma specimens, Ingrid V. et al. developed a new and specific MIP as a sorbent using the MISPE technique. SEM and FTIR were also used to further describe the polymers. Both MIP and NIP were found to have a fine-grained part, irregular surface morphology, and a complex configuration of internal tiny holes at a magnification of 1000, but the MIP structure was found to have larger, more regular, and more available micropores, all of which are good attributes in a sorbent for use in the MISPE process. The FT-IR spectra shows that GCZ-MIP and NIP are chemically the same, but their structures are ordered differently [29].

The objective of this study was to create a novel molecularly imprinted polymer (4-VPMIP) for MA as a solid-phase extraction (SPE) sorbent using a conventional molar ratio of 1:4:20 to achieve optimal imprinting efficiency. The goal was to use the 4-VPMIP in selective urine sample preparation to eliminate the matrix effects [30]. The MIP and non-imprinted polymer (NIP) were characterized using Fourier transform infrared (FI-IR) spectroscopy to identify different functional groups and chemical bonds. The morphologies of the polymers were studied by scanning electron microscopy (SEM). In addition, the proper elution of MA from the MIP was ensured. This study also evaluated the mechanisms of selective adsorption, as well as the thermodynamics and kinetics of adsorption, to determine the specific recognition of the MIP.

## 2. Materials and Methods

### 2.1. Reagents and Solvents

Ultra-pure water was utilized throughout the whole proposed study, and all of the chemicals and solvents used in this research were of the analytical reagent grade. MA (99+%, CAS:90-64-2) was purchased from Thermo Scientific Co.,Ltd. (Shangai, China). EGDMA (98%, CAS NO. 97-90-5) was provided by Merk. KGaA (Darmstadt, Germany), 4-VP (95%, 100 ppm hydroquinone as inhibitor), AIBN solution, and MAA (99%) were bought from Sigma-Aldrich Co. Ltd. (St. Louis, MO, USA). ACN, HPLC-grade was provided by Fisher Scientific Co. (Hampton, NH, USA), and methanol (MeOH, HPLC-grade) was provided by Chem-Lab NV (Zedelgem, Belgium). Acetic acid glacial (AA, CH_3_COOH, 100%) was from PanReac AppliChem (Castellar del Vallès, Spain). Acetone was obtained from Prolabec (Laval, QC, Canada). The Environmental Sciences Department’s industrial waste treatment lab (KAU) has a Millipore purification system with an MPK01 filter from Millipore (Fontenay-sous-Bois, France) that supplies ultrapure water. N_2_ of 10 L steel syl. (99.999%, NG01RTN02) as provided by ASG (KSA) was used to remove O_2_ gas. EGDMA was vacuum distilled to get rid of the inhibitors. In addition, AIBN was recrystallized from MeOH before usage, and 4-VP was purified by running over a straightforward Al_2_O_3_ column to get rid of inhibitors. Urine samples were provided by one of the research team. Morning spot urine samples were collected and kept in sterile containers, at a temperature of −20 °C, until analysis.

### 2.2. Pre-Treatment of Urine

One milliliter of the urine specimen was placed in a test tube, and its pH was adjusted to 2.0 with hydrochloric acid. MA, PGA, and Hip in urine were extracted with 4 mL of a mixture of ethyl ether and methanol (9/1, by vol.). The resulting extract was dehydrated by the addition of anhydrous sodium sulfate. One milliliter of extract was transferred to another test tube, after which approximately 0.2 mL of methanol was added, and the solution was injected into the HPLC.

### 2.3. Analytical Instruments

Analytical separations were performed using high-performance liquid chromatography (HPLC, Agilent Technologies 1200 series, Santa Clara, CA, USA) equipped with a diode array detector (DAD). A digital ultrasonic cleaner (JPS-24AD, 3 L, Moscow, Russia) was used to disperse the mixtures or remove O_2_ from the solution. An oil bath was used to carry out the polymerization. A Fisher Scientific centrifuge (accuSpinTM Micro, Schwerte, Germany) with 24 tube positions was used for better separation of analytes subjected to chromatographic analysis. An IRAffinty-1 Spectroscopy was used to investigate the IR spectra of polymer particles in the range of 4000–400 cm^−1^ (SHIMADZU, Kyoto, Japan). To examine the morphology of polymer particles, a scanning electron microscope (SEM, Quanta 250, Waltham, MA, USA) was used. We used a 250 μL syringe gas chromatographic injector with a sharp tip to inject a micro quantity of MIP components during synthesis (JVLAB, Shanghai, China). MAX 3 mL empty SPE cartridges solid-phase extraction (JVLAB, Shanghai, China) with two frits were used to wash MA from MIPs during the elution step. We used a 0.4 L Laboratory Pulverizer Ball Mill Small Planetary, Ball Grinding Mill Machine (DECO, Hunan Yueyang, China) with compatible grinding jars (ball; PTFE; Teflon) and balls (ZrO_2_; Zirconium oxide) where the minimum granularity of ground MIP can be as small as 0.1 um. A standard test sieve (55 × 28 mm) of 400 mesh stainless steel screen cell strainer (Shijiazhuang, China) with handle was used to obtain a particle size of ≤38 µm. The Environmental Science Department’s Food Safety and Quality Lab has an SPE vacuum manifold (CHROMABOND^®^) of 18 positions that was used to extract the MA throughout the elution process, while the Water Pollution Lab has both a dryer oven (Heraeus, Hanau, Germany) and an analytical balance (Mettler Toledo AL204, Columbus, OH, USA) that were used to dry the polymers and weigh the exact mass of MIP components, respectively.

### 2.4. Synthesis of MA-MIP and NIP

**Figure 2:**
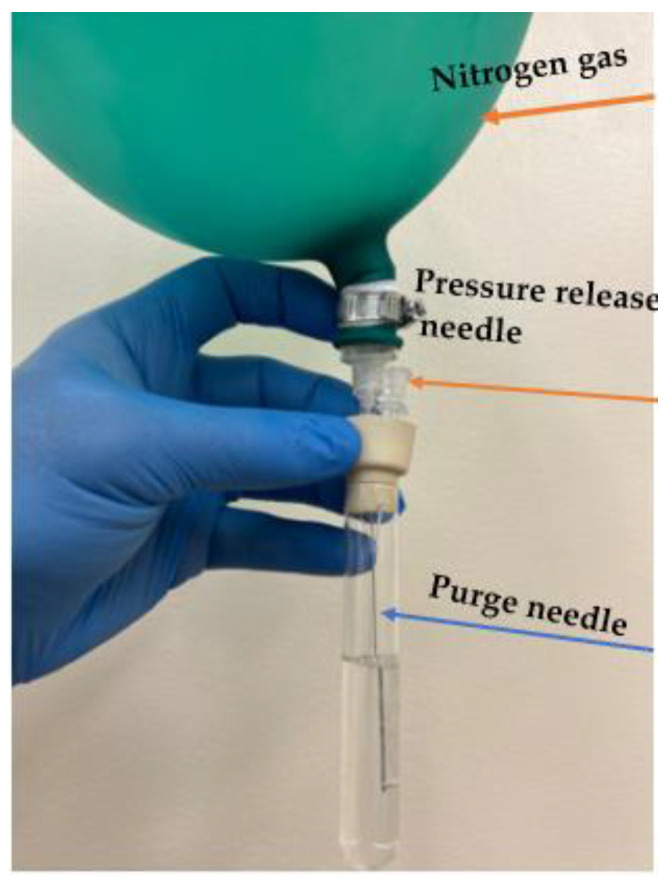
Set up for N_2_ purging with slow to moderate flow rate.

A noncovalent bulk polymerization strategy with a common molar ratio of 1:4:20 was used to generate MIP and NIP for MA. An initial 3 mL of ACN was used to dissolve 1 mmol (0.1522 g) of MA in a 10 mL test tube, and then 4 mmol (427 µL) of 4-VP was added. For 30 min, the mixture was ultrasonicated at room temperature to perform pre-polymerization. To prevent oxidation, the test tube was quickly shut, and the solution was purged with N_2_ to eliminate any dissolved O_2_ as shown in Figure 2.

**Figure 3:**
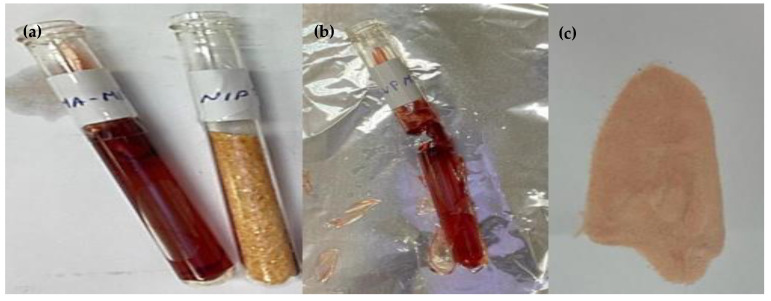
Synthesized MIP and NIP. (**a**) The obtained bulk polymers. (**b**) Crushed vial that contains polymer to be ground. (**c**) The finished MA-MIP powder. The presence of 4-VP as a functional monomer gives polymers their characteristic yellow-brown color.

Subsequently, 20 mmol (3.8 mL) of EGDMA and 2.5 mmol (478 µL) of AIBN were injected. For a further 20 min, the solution was sonicated. To finish the polymerization operation, the resultant reaction mixture was heated in an oil bath at 60 °C for 24 h while being purged with N_2_ gas. The polymer was dried in an oven at 60 °C for 24 h, and then crushed and sieved at room temperature to yield particles that measured 38 µm or less using a test sieve of mesh size 400 (≤38 µm) as shown in Figure 3.

**Figure 4:**
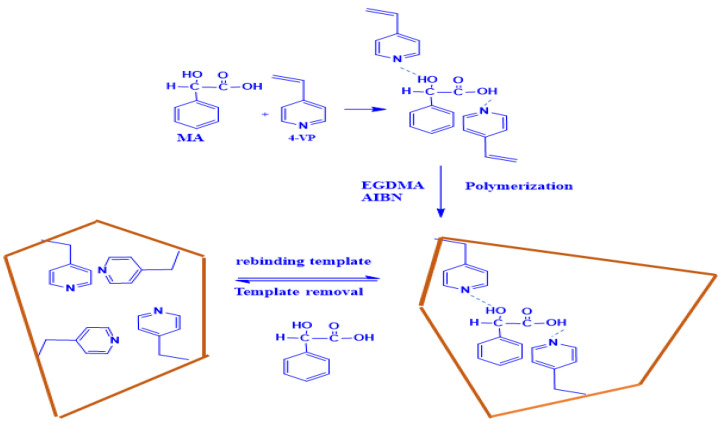
Schematic depicting the synthesis procedures of MA-MIP and the elimination of MA from MIP binding sites.

Figure 4 illustrates the MIP preparation process schematically.

In a similar manner, but without the inclusion of the template molecule, the NIP was produced. The processes used to create MIP and NIP of MA, which have identical compositions of 4-VP and EGDMA throughout bulk polymerization protocol, are shown in Table 1.

### 2.5. MA Elution

**Figure 5:**
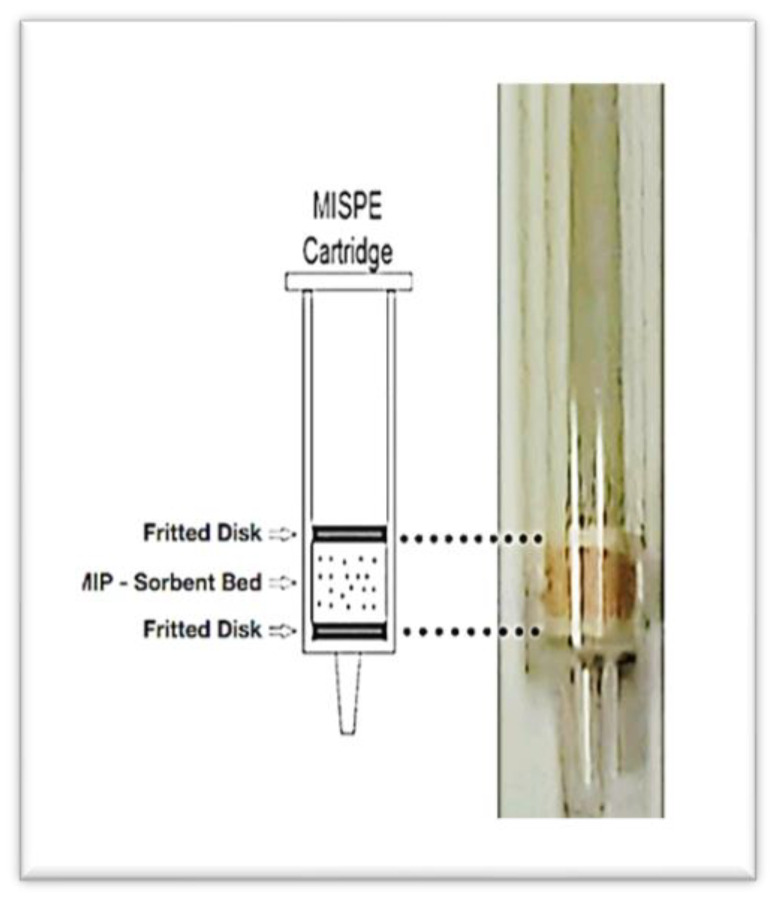
Preparation of 3 mL MISPE cartridge for MA elution process.

The analyte (MA) was eliminated by subjecting MIPs to a series of washes in a solution of MeOH and AA (90:10, *v/v*). The remaining AA was removed from MIP by washing it twice with MeOH, and the powdered MIP was then dried in an oven at 60 °C for 6 h. The 3 mL SPE cartridge was used to complete the elution process including packing around 100 mg of MA-MIP between two polyethylene frits as shown in Figure 5. The packed powder sample was pulled through the stationary phase (9 MeOH:1 AA *v/v*/) using a manifold tool at a regulated extraction rate and sample flow. The sample was then inserted into the HPLC-DAD at 200 nm to measure the peak area of MA in milli-absorbance units (mAUxS) at RT ≈ 7.2 mints. The above procedure was repeated three times in which the MA was no longer detectable.

The mobile phase for the HPLC consisted of ACN, ultra water, and AA in the following proportions: 60:39.5:0.5, *v/v/v*, respectively. The C18 column (250 × 4 mm, 5 μm) was used. The injection volume was set at 20 µL, the flow rate was set at 0.7 mL/min with DAD detection at 225 nm wavelength, and the injection volume was set at 20 μL. The calibration curve showed that the retention time (RT) of MA was 5.17 ± 0.09 min.

### 2.6. Adsorption Experiments

To monitor the amount of MA adsorbed onto the synthesized polymer, HPLC-DAD was employed. Specifically, 2 mg of the polymer was added into 10 mL, with initial concentrations of MA ranging from 10 to 220 mg.L^−1^. These mixtures were shaken at different times (1–60 min), temperatures (293–313 K), and pH values (5–8) followed by centrifugation at 4000 rpm. The resulting supernatant was analyzed using HPLC-DAD to determine the concentration of free MA. The amount of MA adsorbed onto the polymer (*Qe*, mg.g^−1^) was calculated using the following equations:

(1) $$Q_{e}=\frac{\left(C_{0}-C_{e}\right)V}{m}$$

(2) $$Q_{t}=\frac{\left(C_{0}-C_{t}\right)V}{m}$$

where *C*_0_ (mg.L^−1^), *Ce* (mg.L^−1^), and *C_t_* (mg.L^−1^) represent the initial concentration, the equilibrium concentration, and the concentration of MA at each time, respectively. *Qe* (mg.g^−1^) is the adsorption amount, *V* (L) is the volume of the solution, and *m* (g) represents the weight of the polymer.

### 2.7. Adsorptive Selectivity

To carry out selectivity studies, the template desorbed polymer was mixed with solutions of equal volumes. These solutions contained the template (MA) and other styrene metabolites found in urine, such as phenylglyoxylic acid (PGA) and hippuric acid (Hip), each at the same concentration.

**Scheme 1:**
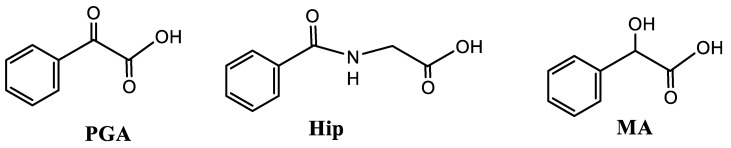
Chemical structure of urinary metabolites.

The extent of binding was then determined using HPLC-DAD, and the differences in binding between the compounds were compared. Scheme 1 illustrates the structural similarities between the selected urine metabolites.

To conduct the experiment, 2 mg of either MIP or NIP was introduced into 10 mL of binary mixtures containing MA/PGA and MA/Hip. PGA and Hip were selected as competitive adsorption molecules and were formed into a binary mixture with MA at the same concentration (200 mg/L) for adsorption under optimal conditions.

The levels of the free analyte and other urine metabolites in the supernatant were analyzed using HPLC-DAD. The equations used to calculate the partition coefficient *Kd* (mL.g^−1^) [31], imprinting factor (*IF*), and selectivity coefficient (α) were as follows:

(3) $$K_{d}=\frac{C_{0}-C_{e}}{C_{0}}\times \frac{V_{s\left(mL\right)}}{\left(Mass\;of\;MIP\;or\;NIP\left(g\right)\right)}$$

(4) $$IF=\frac{K_{d}\left(MIP\right)}{K_{d}\left(NIP\right)}$$

(5) $$\alpha =\frac{IF_{T}}{IF_{M}}$$

where *C*_0_ and *Ce* are the initial and equilibrium concentrations of the analyte, respectively, and *Vs* is the solution volume. *IF_T_* and *IF_M_* are the imprinting factors for the template molecule MA and its metabolites (PGA and Hip).

### 2.8. Solid-phase Extraction Experiments

To prepare a solid-phase extraction column using a molecularly imprinted polymer (SPEMIP), a 3 mL small column was filled with 100 mg of the 4-VPMIP, and polyethylene frits were used to seal both ends of the column. SPEMIP was activated using 3 mL of methanol and 3 mL of water. Following this, the cartridge was loaded with 5 mL of an aqueous solution containing MA at different initial concentrations (ranging from 20 to 100 mgL^−1^) and pH values (between 4 and 8). To elute the target analyte, a mixed solvent of methanol and acetic acid (9:1, *v/v*) was used in a volume of 4 mL. The concentration of the eluate was determined using high-performance liquid chromatography with diode array detection (HPLC-DAD). The extraction efficiency was calculated using the following equation:

(6) $$E\%=\frac{C_{0}-C_{t}}{C_{0}}\times 100$$

where *C*_0_ (mg.L^−1^) and *Ct* (mg.L^−1^) are the concentration of MA before and after extraction, respectively.

### 2.9. Adsorption Kinetics

Two commonly used models, the pseudo-first-order (Equation (7)) and pseudo-second-order kinetic models (Equation (8)), were employed to analyze solid–liquid adsorption and investigate the mechanism of the adsorption process of MIP for MA. The models used are listed in Table 2.

Both the kinetic models discussed above have limitations in accurately describing the diffusion mechanism of MA on SPE-4-VPMIP. To further understand the adsorption mechanism, the intraparticle diffusion and liquid film diffusion models were investigated. Equations (9) and (10) depict the intraparticle diffusion equation and liquid film diffusion equation, respectively:

(9) $$q_{t}=kt^{\frac{1}{2}}+c$$

(10) $$\ln\left(1-F\right)=-k_{f}t$$

where *kd* (g·mg^−1^·min^1/2^) is the rate constant of the intraparticle diffusion model, it can be obtained from the slope of the line *qt*~*t*^1/2^; *c* is constant, *k_f_* is the liquid film diffusion coefficient, min^−1^; *F* = *Q*/*Q_e_* is the adsorption saturation.

### 2.10. Adsorption Isotherm

Adsorption isotherms [32,33,34] play a critical role in the theoretical evaluation and interpretation of thermodynamic parameters as they provide information about the interaction of molecules with adsorbents. These isotherms establish a correlation between the concentration of molecules in the solution and the quantity of ions adsorbed on the solid phase when both phases reach equilibrium [32,33,34,35]. The Langmuir and Freundlich isotherm models were used to study the adsorption performance of MA on SPE4-VPMIP, and the isothermal equations of the two models are expressed by the Equations (11) and (12).

(11) $$\frac{C_{e}}{Q_{e}}=\frac{1}{Q_{m}k}+\frac{C_{e}}{Q_{m}}$$

(12) $$lnQ_{e}=lnk_{f}+\frac{lnC_{e}}{n}$$

where *Ce* is the equilibrium concentration, *Qe* the amount of MA adsorbed at equilibrium, *Qm* is amount of MA adsorbed for a complete monolayer, *k* is a constant related to the energy or net enthalpy of sorption. The Freundlich adsorption model employs *K_f_* and *n* as isotherm constants [33,34,36]. To assess the suitability of the Freundlich model, a plot of log *Q_e_* against log *C_e_* was created and *K_f_* and *n* were derived from the plot. In our MIP systems, both Langmuir and Freundlich isotherms were employed, and their outcomes were compared to ascertain the appropriate isotherm for our system.

## 3. Results and Discussion

### 3.1. Characterization of Physical Properties of 4-VPMIP and NIP

#### 3.1.1. Scanning Electron Microscopy (SEM)

**Figure 6:**
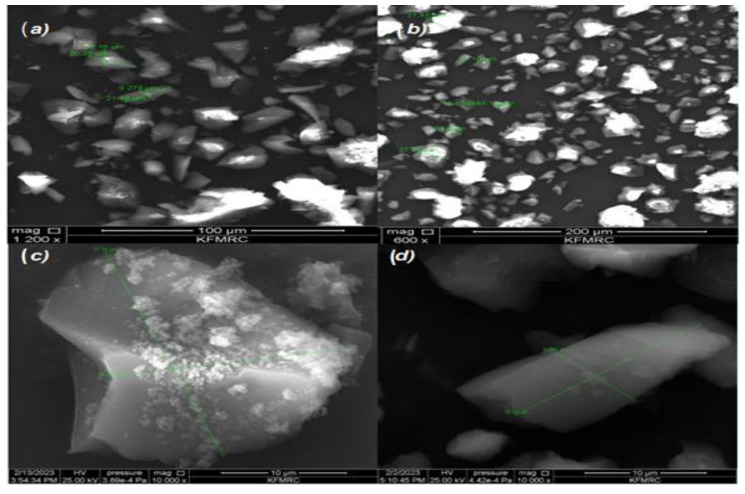
SEM images of MA-MIP and NIP sizes at different magnifications: (**a**) ×1200; (**b**) ×600; (**c**,**d**) ×10,000.

The form and size of polymer particles may be determined from an SEM investigation, making it a crucial tool in morphological research. Figure 6 shows that µm-sized irregular particles are generated. This is due to the fact that bulk polymerization was used in the synthesis of the polymer particles [37]. These microscopic particles provide extensive template–MIP surface contact, which ultimately results in a high concentration of MA.

**Figure 7:**
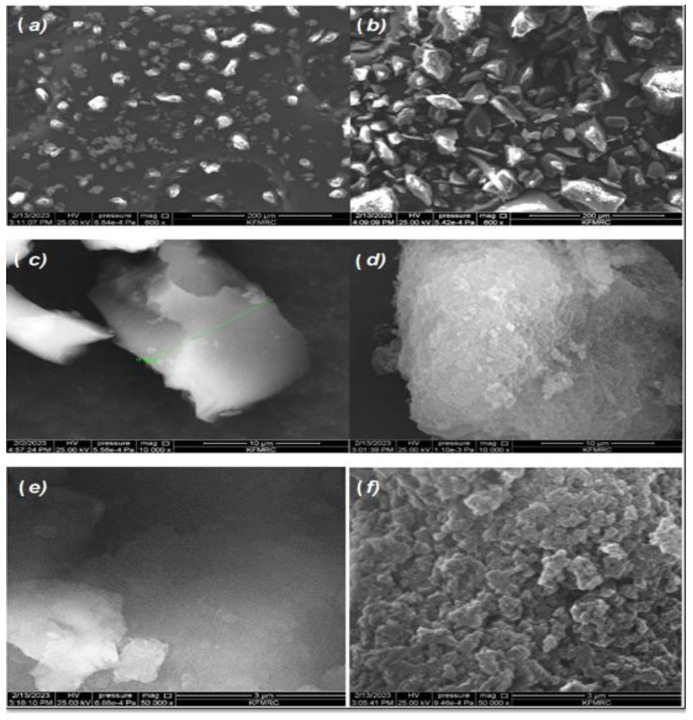
SEM images of (**a**,**c**,**e**) NIP and (**b**,**d**,**f**) MA-MIP, at a magnitude of 600×, 10,000× and 50,000×, respectively.

As can be seen in Figure 7, the presence of the template molecule in the imprinting process is responsible for the difference in particle size that exists between the MIP and NIP particles. The addition of MA to the reaction medium led to an increase in the formation and expansion of the particle surfaces, which allowed the MIP particles to become more sizeable. Moreover, MIP surfaces are rougher and have a higher porosity structure than NIP surfaces as a result of the elution of the MA compounds in the MIPs, which left a huge range of cavities. This is proof that the MA was effectively removed from MIP, leaving its imprint on the polymers when MA was used as the molecular template and 4-VP was used as a functional monomer to prepare MIP particles by bulk polymerization [38]. In fact, MA-MIP adsorbs analytes (MA) significantly better than NIP due to the MA-MIP particles’ pores, which led to a greater surface area than that of NIP [16]. While the imprinted holes could not be seen because of the MA molecule’s tiny size, differences seen in SEM images would suggest the existence of MA molecules during bulk polymerization, leading to a distinct polymeric development in terms of structure [39]. All the polymers that have been made seem to be hard. These morphologically unique characteristics may indicate that the MIP and NIP were effectively created [40].

#### 3.1.2. FT-IR Spectroscopy

**Figure 8:**
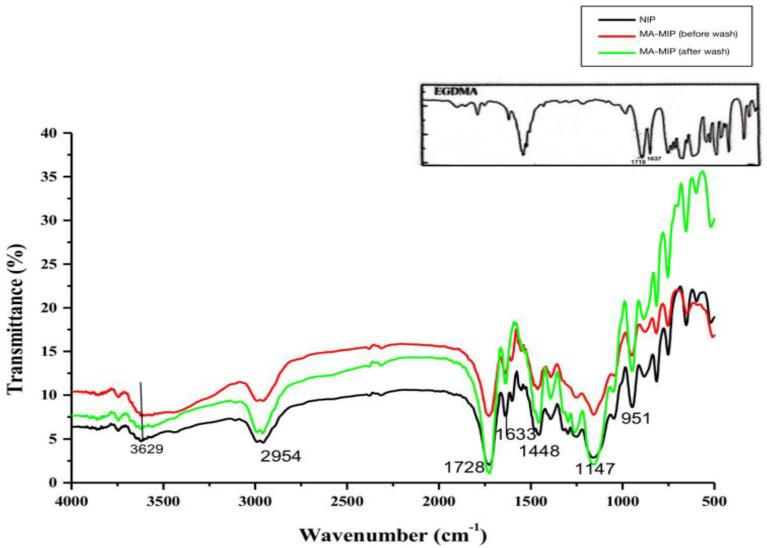
FT-IR spectra of NIP (Black) and MIP before (Red) and after (Green) MA removal.

Polymeric materials can change in their molecular structure or composition when they are mixed, heated, or exposed to light, among other things. These updates can be completed in a matter of seconds, or they can take several hours. Figure 8 shows the IR spectra used to find out what functional groups were in the MIP before (a) and after elution (b), as well as in the NIP (c), and to ensure the interaction among MA, 4-VP, and EGDMA. The presence of MA as an analyte in the matrix material of MIPs causes the IR spectra of MIPs with MA (before wash) to be semi-similar to those of NIP, while the spectra of MIP (after wash) showed compositions and absorption peaks that were very similar to those of NIP, demonstrating that all MA were totally eliminated after the extraction phase [41]. For all polymers, the polymerization was confirmed by the presence of C-O-C and C=O bounds of EGDMA, which caused the bands at 1147 cm^−1^ and 1728 cm^−1^ to be seen. The EGDMA characteristic peaks were observed by Can Zhou et al. at 1718 cm^−1^ for C=O and 1637 cm^−1^ for C=C stretching vibrations [42]. In contrast, the IRs of MIP (before wash), MIP (after wash), and NIP show stronger C=O stretching vibrations at 1728 cm^−1^ and weaker C=C stretching vibrations at 1633 cm^−1^. This demonstrates that EGDMA has been effectively cross-linked among the polymer monoliths. Moreover, around 2954 cm^−1^ to 2995 cm^−1^, the aliphatic compound’s C-H stretching vibration mode may be seen. There are two peaks that are indicative of 4-VP: a stretching vibration peak at 1633 cm^−1^ due to the C=N functional group in the aromatic ring and a very weak signal due to the C=C functional group in the vinyl at 1521 cm^−1^. The results demonstrate that 4-VP was successfully polymerized into the MIPs. The presence of the template, which formed hydrogen bonds with 4-VP, probably caused a relatively broad peak at 3629 cm^−1^ compared to the NIP and MIP infrared spectra (after washing) [43]. Therefore, the results from the FT-IR spectra suggest that the MIPs were properly synthesized.

### 3.2. Effect of Initial Concentration on Adsorption Capacity

**Figure 9:**
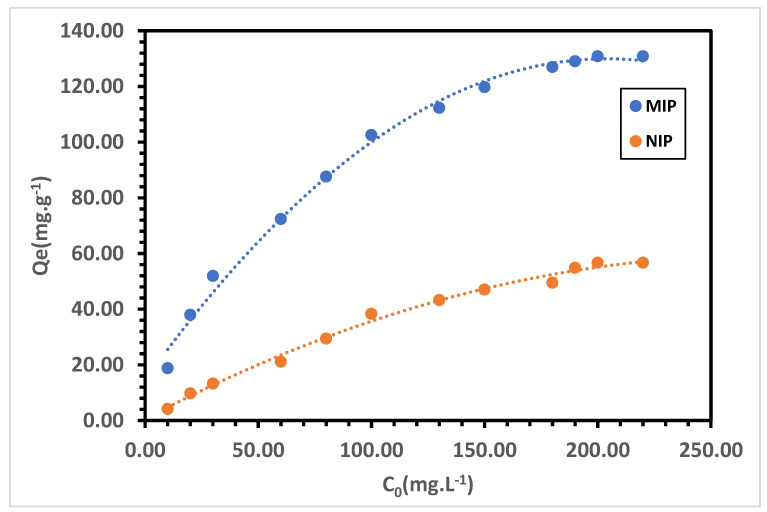
Adsorption capacity studies for the 4-VPMIP and NIP.

Equilibrium binding analysis was conducted to observe the binding performance of surface-imprinted 4-VPMIP compared to that of the control NIP. The functional monomer 4-VP and template molecule MA interact through hydrogen bonding and π–π interactions. The binding isotherm displayed a saturation curve, indicating the presence of a finite number of binding sites in the imprinted polymer. As shown in Figure 9, the adsorption capacity of 4-VPMIP was significantly higher than that of NIP, demonstrating MIP’s unique capacity of the 4-VPMIP for MA, which can be attributed to the complementary cavities present in the 4-VPMIP materials. The 4-VPMIP imprinted surface exhibited a greater binding capacity than the NIP. The weak adsorption of MA to the NIP was likely due to a non-specific interaction with the polymer matrix. Figure 9 illustrates that the adsorption capacity of the 4-VPMIP increased with the initial concentration of MA. Specifically, the adsorption amount of 4-VPMIP went up from 18.79 mg.g^−1^ to 130.84 mg.g^−1^ as the concentration of MA increased from 10 mg.L^−1^ to 200 mg.L^−1^. However, the adsorption amounts of the 4-VPMIP did not show significant changes when the concentration of MA was increased beyond 200 mg.L^−1^. This is because the number of imprinted pores on the surface of the MIP remains constant, and as the concentration of MA increases, the imprinted pores gradually become occupied and saturated. The adsorption amount of 4-VPMIP reached its maximum when the concentration of MA was 200 mg.L^−1^. As a result, 200 mg.L^−1^ was determined as the optimal concentration of MA in the subsequent experiments.

### 3.3. Effect of pH on Adsorption Capacity

Several factors affect the strength of the hydrogen bond between mandelic acid, which is the target molecule, and pyridine, which is anchored on the polymeric support. These factors include the distance and orientation of the functional groups, solvent polarity, and temperature. Mandelic acid and pyridine have hydrogen bond donors and acceptors, respectively, in their structures. When in close proximity, they can form hydrogen bonds between their functional groups. The interaction between pyridine and the hydroxyl groups is primarily governed by hydrogen bonding. The effect of pH on this interaction bond can be significant because the protonation state of these functional groups can change with the pH.

**Figure 10:**
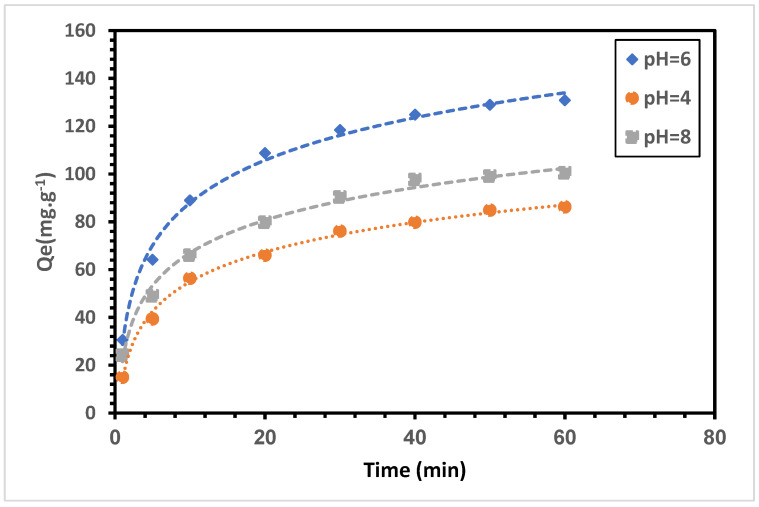
Effect of pH on adsorption capacity of MIP(4-VP).

Figure 10 demonstrates that the pH of the solution does not have a significant impact on the duration required for the 4-VPMIP to attain adsorption equilibrium. Based on our experimental results, when the solution pH was 6, the adsorption capacity of the 4-VPMIP rapidly increased to 130.84 mg.g^−1^ within 60 min and then reached its maximum value. Furthermore, as time progressed, the number of imprinted sites on the polymer surface declined, leading to a reduction in the adsorption rate of the target molecule, ultimately resulting in adsorption equilibrium being reached after 60 min. Consequently, 60 min was chosen as the optimal adsorption time to enhance the adsorption efficacy of the 4-VPMIP.

At pH 6, the carboxylic acid group of mandelic acid (pKa~3.5) is completely deprotonated (-COO-), whereas the phenolic groups of MA are in their protonated state (pKa~9.8). In contrast, the pyridine molecule is in a neutral state (pKa~5.25). Under these conditions, the interaction between mandelic acid and pyridine was mainly driven by the formation of hydrogen bonds between the partially negative oxygen atom of the carboxylate group in mandelic acid and the partially positive nitrogen atom in the pyridine ring. When the adsorption time was 60 min and the MA solution changed from pH = 6 to pH = 8, the adsorption amount of MIP decreased from 130.84 mg.g^−1^ to 100.46 mg.g^−1^. At pH values higher than 6, the carboxylic acid group in mandelic acid is mostly deprotonated and in its anionic form (-COO-), while the pyridine molecule is mostly in its neutral state. Under these conditions, the interaction between mandelic acid and pyridine is primarily driven by the formation of weak hydrogen bonds between the partially negative oxygen atom of the carboxylate group in mandelic acid and the partially positive nitrogen atom in the pyridine ring. Therefore, at pH > 6, the interaction between mandelic acid and pyridine is weak.

At pH = 4, both mandelic acid and pyridine will be predominantly in their protonated form. At this time, the adsorption amount of 4-VPMIP is reduced to 86.21 mg.g^−1^. At this time, the adsorption capacity of 4-VPMIP is relatively strong, but when the solution changes from neutral to weakly alkaline, the adsorption amount of 4-VPMIP is reduced to 86.21 mg.g^−1^. The prevailing factor in the interaction between mandelic acid and pyridine under these conditions is the creation of a salt bridge. This occurs between the positively charged pyridinium cation (C_5_H_5_NH^+^) and negatively charged carboxylate anion (C_8_H_7_O_2_^−^) of mandelic acid. The current conditions did not promote the adsorption of MA by 4-VPMIP. As a result, for the following experiments, a pH of 6 was determined as the optimal condition.

### 3.4. Effect of Temperature on Adsorption Capacity

**Figure 11:**
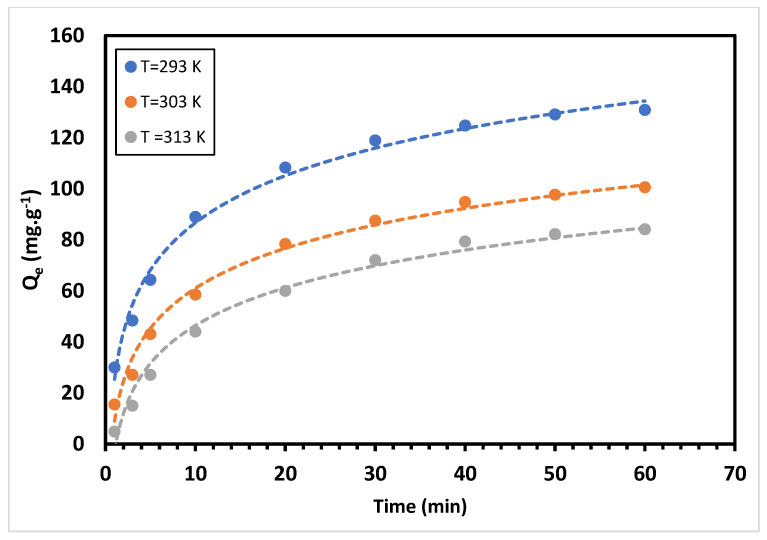
Effect of temperature on adsorption capacity of MIP(4-VP).

The graph in Figure 11 demonstrates that as the temperature increased, the Qe of MIP(4-VP) decreased, suggesting that the adsorption process was exothermic in nature. This performance was ascribed to the hydrogen bond interaction between the adsorbent and MA, which weakened at higher temperatures owing to the weak hydrogen bond force. Furthermore, variations in the external temperature achieved within 60 min did not noticeably affect the time required for the 4-VPMIPMIP to attain adsorption equilibrium. With a duration of 60 min for the adsorption process, increasing the temperature from 293 to 303 K resulted in a reduction of 30.30 mg.g^−1^ in the adsorption capacity of the 4-VPMIP. Nonetheless, as the temperature was further increased to 313 K, the adsorption capacity dropped by 16.44 mg.g^−1^ relative to that at 303 K, indicating that the temperature has a relatively notable impact on the adsorption capacity. This is because, as the temperature rises, the imprinted sites on the imprinted materials are disrupted, leading to the collapse of the imprinted pores and a decrease in the adsorption capacity. Thus, 293 K was chosen as the optimal temperature for adsorption.

### 3.5. Adsorptive Selectivity

To explore the selective adsorption capacity of 4-VPMIP for mandelic acid (MA), a binary mixed solution containing phenylglyoxylic acid (PGA) and hippuric acid (Hip), both of which are urine metabolites, was created to serve as a competitive adsorption molecule. The distribution coefficient Kd (mL.g^−1^), imprinting factor IF, and relative selectivity coefficient (α) were computed using Equations (3)–(5) outlined in the Experimental section. The results of the cross-selectivity assessment of MIP are presented in Table 3, indicating a successful imprinting effect on the polymer and its selectivity. The greater selectivity for MA can be justified by the following findings: Initially, the distribution coefficients of MA in the 4-VPMIP were higher compared to the interferents, with values of 654.18 vs. 219.25 for PGA and 654.18 vs. 154.25 for Hip. Second, the imprinting factor (IF) values for MA were higher in MIP than those for the interferents, which reveals that MIP possesses a higher degree of selectivity and affinity for the target molecule MA. Third, the relative selectivity coefficient α values for the binary mixtures of MA/PGA and MA/Hip were 2.91 and 4.21, respectively; both were greater than 1, indicating that the 4-VPMIP exhibits an excellent affinity for MA adsorption.

### 3.6. Adsorption Kinetics and Isotherms

#### 3.6.1. Adsorption Kinetics

**Figure 12:**
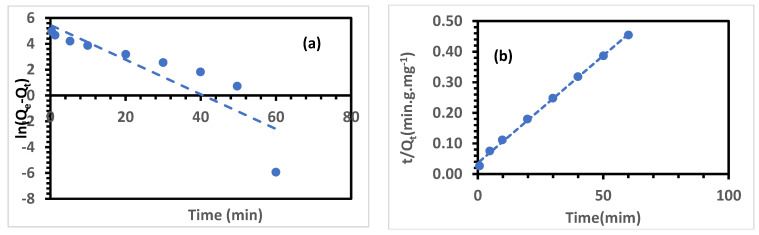
Fitting curves of pseudo-first-order (**a**) and pseudo-second-order models (**b**).

**Figure 13:**
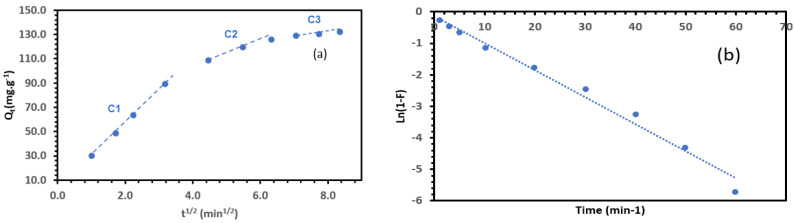
Fitting curves of intraparticle diffusion (**a**) and liquid film diffusion models (**b**).

Kinetic equations were utilized to fit the experimental data, and the corresponding fitting graphs and parameters are presented in Table 4 and Figure 12, respectively. The comparison between Figure 12a,b reveals that the pseudo-second-order kinetic model provides a more suitable description of the adsorption process than the pseudo-first-order kinetic model. The correlation coefficient ($R_{2}^{2}$) of the pseudo-second-order kinetic model was 0.9980, which was higher than that of the pseudo-first-order kinetic model ($R_{1}^{2}$). These results suggest that the binding of MA to 4-VPMIP is primarily controlled by chemisorption rather than by the material transport step. Figure 13 displays a linear representation of the intraparticle diffusion model for MA adsorption onto the 4-VPMIP.

Table 5 presents the coefficients obtained from the intraparticle and liquid film diffusion equations. The graphical representation in Figure 13 shows that the intraparticle diffusion model of the 4-VPMIP does not originate from the origin, suggesting that the adsorption process is not a single-step mechanism. The initial rapid adsorption stage occurred within the first 9 min, followed by a slow adsorption stage between 20 and 40 min, which was controlled by both intraparticle diffusion and liquid film diffusion modes. The third stage of adsorption, the equilibrium stage, occurred between 50 and 70 min. This is evident from the gradual reduction in kd_1_, kd_2_, and kd_3_ in Table 5. Furthermore, it can be observed from the gradual increases in c_1_, c_2_, and c_3_ (Figure 13) that the impact of the liquid film diffusion model becomes progressively more significant with time.

#### 3.6.2. Adsorption Isotherm

**Figure 14:**
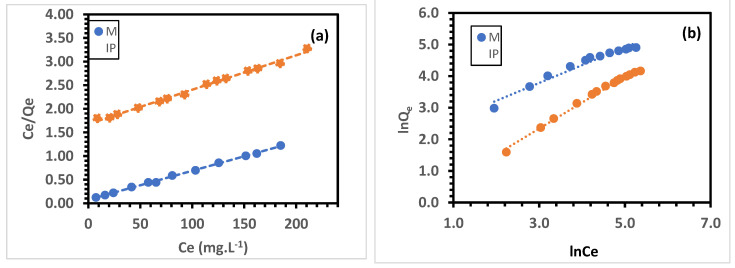
Linear fit plot of Langmuir (**a**) and Freundlich (**b**) isotherm models.

Isothermal adsorption experiments were conducted to investigate the adsorption capacities of 4-VPMIP and NIP. As shown in Figure 14 and Table 6, it is evident that the Langmuir model provides a better fit than the Freundlich model. The linear correlation coefficients obtained from the Langmuir isotherm equation were 0.9983 and 0.9943 for the adsorption of MA on the 4-VPMIP and NIP, respectively, while those from the Freundlich isotherm equation were relatively small. Hence, the adsorption behavior of MA on 4-VPMIP and NIP can be effectively described by the Langmuir isotherm model, which assumes that the adsorption process occurs through a limited number of adsorption sites with identical properties. Furthermore, the values of 1/n obtained from the Freundlich isotherm model for the adsorption of MA on 4-VPMIP and NIP were 0.5627 and 0.9926, respectively, indicating that the adsorption process is a typical single-molecule adsorption process.

### 3.7. Reusability of SPE-4-VPMIP

**Figure 15:**
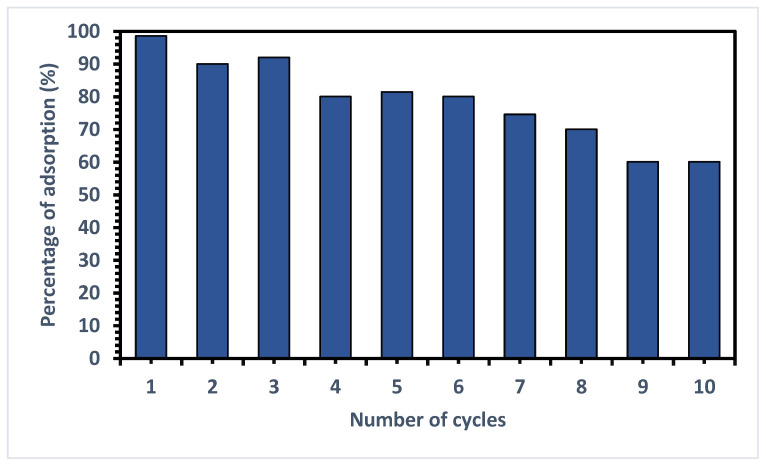
SPEMIP adsorption efficiency for MA after 10 regeneration cycles.

Ten complete cycles were used to evaluate the reusability of the 4-VPMIP cartridges. Between cycles, the MIP cartridge was reconditioned with methanol/acetic acid (9:1, *v/v*) until MA was undetectable in the filtrate. Figure 15 shows the adsorption efficiency of SPE4-VPMIP over 10 consecutive adsorption–desorption cycles with an initial MA concentration of 100 ppm. This test was critical for demonstrating the possible reusability of SPE4-VPMIP. The results showed that SPE4-VPMIP displayed a constant efficiency over six adsorption–desorption cycles, with the removal efficiency maintaining 81% of the initial efficiency after six cycles (Figure 15). The efficiency decreased in cycle 9, reaching 60%. This decrease in uptake may be due to the progressive saturation of 4-VPMIP active sites, preventing more template uptake.

### 3.8. Application of the Proposed SPE4-VPMIP to the Extraction of MA from Human Urine with HPLC-DAD

To evaluate the applicability of the developed SPE4-VPMIP for the extraction of MA from urine samples provided by one of our research team’s researchers, the SPE4-VPMIP-HPLC-DAD procedure was employed for detecting MA in spiked urine samples. Urine samples were spiked with MA at 50, 100, and 150 mg.L^−1^. Table 7 shows the results obtained for each sample after extraction by SPE4-VPMIP. The urine samples were analyzed in triplicate, and the results are presented in Table 7. The recovery of MA concentrations in the spiked urine samples was within 96–98% with %RSD < 5.2.

**Figure 16:**
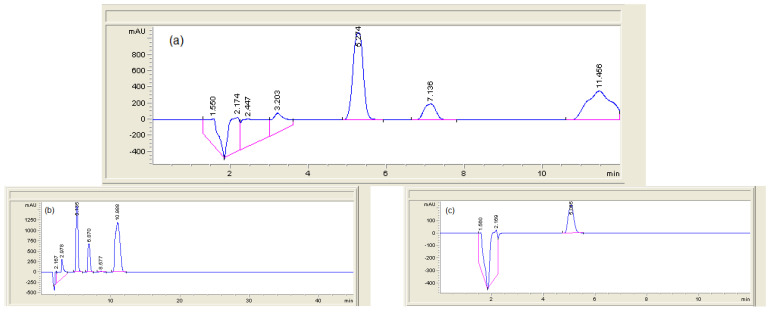
HPLC chromatograms of a urine sample spiked with MA (**a**), a standard mixture of urinary metabolites (**b**), and desorption of MA from a spiked urine sample after MIMEPS extraction (**c**).

Because of the specificity of the extraction provided by 4-VPMIP, very clean chromatograms were obtained, thus allowing for a lower detection limit. Figure 16 shows a chromatogram of a urine sample spiked with MA at 50 μg/mL (a), a standard mixture of urinary metabolites (b), and desorption of MA from a spiked urine sample after MIMEPS extraction (c). No interfering peaks were detected at the retention time of MA when the standards and quality control samples were analyzed. Moreover, in the presence of interferents (PGA and Hip), as explained in the study of the imprinting effect and selectivity of the 4-VPMIP section, there was no overlap in the chromatogram (Figure 16c), indicating that MIMEPS had good selectivity and could eliminate interfering compounds while enriching MA to a sufficient level. These results also imply that MA in human urine is quantifiable. The ability of our polymer to be selectively extracted in only one step allows for increased sensitivity. Additionally, initial testing suggests that these polymers could be used for the exclusive extraction of MA in other biological fluids.

## 4. Conclusions

In our study, for the first time, a new sample preparation technique using the combination of MIP and SPE was introduced for the analysis of MA in human urine. In this method, bulk polymerization protocol and a noncovalent approach, which are the most widely used, were applied in the synthesis of MIPs under the optimum mole ratio of 1:4:20 using MA as templates with 4-VP functional monomers and EGDMA cross-linking agent. The synthesized MIP was characterized by two distinctive techniques (FTIR spectra and SEM) showing morphological and physicochemical characteristics suitable to be used as sorbent in SPE, especially the presence of permanently porous microparticles that can be applied as sorbents for effective removal and preconcentration of MA from a complex sample. The adsorption kinetic study showed that the adsorption process of 4-VPMIP was well fitted by the pseudo-second-order model. The isotherm was thermodynamically feasible and could occur spontaneously, and the equilibrium adsorption data were well fitted by the Freundlich model. The selective recognition and enrichment experiment indicated that 4-VPMIP could be used to enrich MA from the urine sample. The findings show that MIPs were synthesized successfully, and the MA was removed completely. The method we used is simple, inexpensive, and both user-friendly and environmentally friendly. It could be used for the analysis of MA as a biomarker of exposure to styrene in a complex matrix such as urine.

## Figures and Tables

**Table 1:** Synthesis of 4-VPMIP and NIP for mandelic acid (MA) by bulk polymerization protocol.

Code	4-VPMIP	NIP
Template (mmole)	MA (1)	--------
Functional Monomer (mmole)	4-VP (4)	4-VP (4)
Cross-linker (mmole)	EGDMA (20)	EGDMA (20)
Porogen (mL)	ACN (3 mL)	ACN (3 mL)

**Table 2:** Equations of the kinetic models for the theoretical analysis of sorption data.

Equation *	Definition	Equation Number
$\ln\left(Q_{e}-Q_{t}\right)=lnQ_{1cal}-k_{1}\;t$	Pseudo-first-order model (PFO)	(7)
$\frac{t}{Q_{t}}=\frac{1}{k_{2}\left(Q_{2cal}\;\right)}+\frac{t}{Q_{2cal}}$	Pseudo-second-order model (PSO)	(8)

* *Qe* and *Qt* (mg.g^−1^) amount of MA sorbed at equilibrium and at time *t* (min), respectively; *Q_*1*_cal* and *Q_*2*_cal* are the adsorption capacity calculated according to the pseudo-first-order kinetic equation and the pseudo-second-order kinetic equation, respectively; *k*_l_—rate constant of the PFO kinetic model (min^−1^); *k*_2_—rate constant of the PSO kinetic model (g.mg^−1^.min^−1^).

**Table 3:** Selectivity parameters of 4-VPMIP (distribution coefficient (Kd), imprinting factor (IF), and relative selectivity coefficient (α)).

Target and Metabolites Solution	Polymer	Kd (mL/g)	IF	αT/M	
				αMA/PGA	αMA/Hip
MA	MIP	654.18	2.31	2.91	4.21
	NIP	283.60			
PGA	MIP	219.25	0.79		
	NIP	276.75			
Hip	MIP	154.25	0.54		
	NIP	281.75			

**Table 4:** Parameters of pseudo-first-order and pseudo-second-order models.

Qexp (mg.g^−1^)	Pseudo-First-Order Kinetic Model			Pseudo-Second-Order Kinetic Model		
	*Q*1,*cal*	$R_{1}^{2}$	*k*1 (min^−1^)	*Q*2,*cal*	$R_{2}^{2}$	*k*2 (g.mg^−1^.min^−1^)
130.84	234.042	0.7754	0.134	142.857	0.998	1.357.10^–3^

**Table 5:** Parameters of intraparticle diffusion and liquid film diffusion models for MA adsorption on the 4-VPMIP.

Intraparticle Diffusion Model						Liquid Film Diffusion Model	
K_d1_	K_d2_	K_d3_	c_1_	c_2_	c_3_	k_f_	Intercept
27.353	9.255	2.296	2.149	67.899	112.8	0.086	0.138

**Table 6:** The parameters of the Langmuir and the Freundlich isotherm models for 4-VPMIP and NIP.

Polymer	Langmuir Parameters			Freundlich Parameters		
	Q_m_ (mg.g^−1^)	k (L.mg^−1^)	R^2^	1/n	k_f_ [(mg.g^−1^)^1/n^]	R^2^
MIP	163.93	0.0821	0.9983	0.5627	8.1327	0.9631
NIP	136.98	0.0043	0.9943	0.9926	0.8814	0.9926

**Table 7:** Efficiency of SPEMIPS urine samples spiked with 50, 100, and 150 mg/L of MA.

Spiked Urine (mg.L^−1^)	Recovery (%)	RSD (%)
50	96	4.8
100	98	5.1
150	98	4.5
